# Antiproliferative activity of hexane extract from Tunisian *Cistus libanotis*, *Cistus monspeliensis* and *Cistus villosus*

**DOI:** 10.1186/1752-153X-7-47

**Published:** 2013-03-05

**Authors:** Mariem Ben Jemia, Mohamed Elyes Kchouk, Felice Senatore, Giuseppina Autore, Stefania Marzocco, Vincenzo De Feo, Maurizio Bruno

**Affiliations:** 1 Laboratoire des Plantes Extremophiles - Biotechnologic Center Borj-Cedria Technopark, B.P. 901, 2050, Hammam-Lif, Tunisie; 2 Department of Pharmacy, University of Naples “Federico II”, Via D. Montesano, 49-80131, Naples, Italy; 3 Department of Pharmacy, University of Salerno, via Ponte Don Melillo, 84084, Fisciano (Salerno), Italy; 4 Department STEMBIO, Sect. of Organic Chemistry, University of Palermo, Viale delle Scienze, Parco d’Orleans II, 90128, Palermo, Italy

## Abstract

**Background:**

As a part of our investigation on Tunisian medicinal plants, we have carried out a phytochemical investigation of the hexane extracts from leaves of *Cistus libanotis*, *C. villosus* and *C. monspeliensis*, evualuating also their possible antiproliferative activity *in vitro.*

**Results:**

The major compounds of hexane extracts were identified and quantified by GC-MS. The composition of the three species, although belonging to the same genus, is completely different. The antiproliferative activity was evaluated against murine monocyte/macrophages (J774.A1), human melanoma cells (A-375), and human breast cancer cells (MCF-7), showing major activity against the human melanoma cell line A-375.

**Conclusions:**

The chemical composition of the hexane extracts from the three *Cistus* species can be useful in the chemosystematics of this complex genus. The preliminary antiproliferative activity against human melanoma cell line A-375 deserve further investigations in order to determine the compounds, or their combinations, which are the main responsible for the antiproliferative activity and its possible mechanism(s) of action.

## Background

Cistaceae is a Mediterranean native family of almost 200 species of shrubs. Most members of this family are very fragrant and sweet smelling, being much appreciated in the perfume industry and for ornamental purposes. Also, Cistaceae plants adapt easily to wildfires that destroy large forest areas, their seeds resisting and repopulating rapidly in the following season [1]. This family is formed by different genera, including *Helianthemum*, *Halimium* and *Cistus*. This latter contains between 16 and 28 different species, depending on the source [2]. Some of the *Cistus* species are endemic and others are widespread in the Iberian Peninsula, Canary Islands, Northwestern Africa, Italy, Greece and Turkey [3]. The species are disseminated over different areas of the Mediterranean area, but not all the species are distributed following the same pattern. Thereby, each area is colonised by different *Cistus* species depending on climatological and soil conditions.

Traditional folk medicine has used *Cistus* species as antiinflammatory, antiulcerogenic, wound healing, antimicrobial, cytotoxic and vasodilator remedies. Recent studies highlighted some information on the possible candidate compounds for these effects, and new activities are being discovered and attributed to *Cistus* extracts. These include antimicrobial, antioxidant, antiproliferative, antinociceptive and analgesic effects [4-6].

A comprehensive study on the qualitative composition of the hexane extract of *C*. *monspeliensis* L. leaves has been reported [7] as well as the catechin related compounds in aqueous extracts of the same species [8]. The composition of aqueous extracts from *Cistus libanotis* L. has also been reported [9]. No previous reports on the composition of *C. villosus* L. are available.

Here we present a comparative qualitative and quantitative study of the composition of hexane extracts from the aerial parts of three *Cistus* species grown in Tunisia. Hexane extracts from *Cistus monspeliensis*, *C*. *libanotis* and *C. villosus* leaves were analyzed by GC-MS. Moreover, the antiproliferative activity against a panel of cancer cell lines has been evaluated.

## Results and discussion

### Chemical composition of hexane extracts

As a part of our investigation on Tunisian medicinal plants, we have conducted a phytochemical investigation of the hexane extracts of *Cistus libanotis*, *Cistus villosus* and *Cistus monspeliensis*, evaluating also their possible antiproliferative activity against murine monocyte/macrophages (J774.A1), human melanoma cells (A-375), and human breast cancer cells (MCF-7). The composition of the three hexane extracts was achieved by GC-MS. Although the three species belong to the same genus, the composition of their hexane extract is completely different (Table 1). A total of 47 constituents, representing 90.1% of the total extract, have been identified from the hexane extract from the leaves of *C. libanotis*. In Table 1, the retention indices, retention times and percentage composition are given; the components, grouped in class of substances, are listed in order of elution on a HP 5MS column. By far flavonoids (30.2%) were the main fraction of the extract, with quercetagetin 3',4',6,7-tetramethyl ether (24.6%) as the principal compound. The main component of fatty acids fraction (24.4%) was (*Z,Z,Z*)-9,12-15-octadecatrienoic acid (23.5%). Monoterpene hydrocarbons were also present in good amount (10.5%), being camphene (3.6%) and β-pinene (3.0%) the principal compounds. Hydrocarbons and oxygenated monoterpenes represented 9.3% and 7.1%, respectively. Diterpenes (2.2%), sesquiterpenes (1.3%) and oxygenated sesquiterpenes (0.7%) were present in small amount. In *C. monspeliensis* (36 compounds) extract the principal class was represented by fatty acids (43.3%) among which the most abundant were (*Z,Z,Z*)-9,12-15-octadecatrienoic acid (14.7%) and (*Z,Z*)-9,12-octadecadienoic acid (6.6%). Hydrocarbons (22.8%) are also present in good amount with nonacosane (7.1%) and heptacosane (6.1%) as principal ones. Vitamin E was present (11.5%) in higher amount with respect to *C. libanotis* (3.3%). Monoterpenes, oxygenated monoterpenes, diterpenes and triterpenes were present in quite low amount. The peculiar characteristic of the composition of the extract of *C. villosus* is the high quantity of hydrocarbons (37.3%), being nonacosane (18.3%) and hentriacontane (9.2%) the main compounds. Fatty acids (10.6%) and diterpenes (4.8%) were also present in good amount. It is noteworthy the good quantity of vitamin E (22.7%), the most abundant products among the 31 compounds of the extract of *C. villosus.*

**Table 1:** **Percentage composition of the hexane extract from aerial parts of three *Cistus *spp**

**Component**	**K**_**i**_	**% CL**	**% CM**	**% CV**
**Hydrocarbons**		**9.3**	**22.8**	**37.3**
Tricosane	2300		t	0.1
Pentacosane	2500	2.4	1.8	3.1
Heptacosane	2700	2.2	6.1	6.6
Octacosane	2800	0.3	3.6	t
Nonacosane	2900	3.3	7.1	18.3
Hentriacontane	3100	1.1	4.2	9.2
**Carbonylic compounds**			**0.7**	**2.2**
Undecan-2-one	1287		0.7	2.2
**Monoterpene hydrocarbons**		**10.5**	**2.1**	**0.9**
Tricyclene	928	0.1		
α-Thujene	931	0.1		
α-Pinene	938	2.1		
Camphene	953	3.6		
Sabinene	973	1.2		
β-Pinene	980	3.0		
α-Terpinene	1012	0.3		
*p*-Cymene	1025	0.1	0.8	0.3
Limonene	1030		1.3	0.6
**Sesquiterpene hydrocarbons**		**1.5**	**t**	
Cyclosativene	1363	t		
α-Copaene	1377	t		
α-Elemene	1398	0.2		
β-Caryophyllene	1414	0.7		
Widdrene	1433		t	
γ-Elemene	1438	t		
α-Humulene	1455	0.1		
*allo*-Aromadendrene	1463	0.4		
1-S-*cis*-Calamenene	1520	0.1		
**Oxygenated monoterpenes**		**6.6**	**2.0**	**1.1**
1,8-Cineole	1034	0.1	1.1	0.4
*cis*-Sabinene hydrate	1063	t		
*trans*-Sabinene hydrate	1086	0.7		
α-Campholenal	1128	t		
Camphor	1145	0.7		
Borneol	1167	1.2		
Myrtenol	1197	0.3		
Linalyl acetate	1277	0.7	0.9	0.7
Isobornyl acetate	1284	0.3		
Bornylacetate	1286	1.8		
α-Terpenyl acetate	1343	0.8		
**Oxygenated sesquiterpenes**		**0.7**	**1.4**	**1.2**
Globulol	1587	t		
Viridiflorol	1593	0.4	1.4	1.2
Caryophylla-4(12),8(13)-dien-5β-ol; Caryophylladienol I	1640	0.2		
Caryophylla-3,8(13)-dien-5-β-ol	1649	0.1		
**Diterpenes**		**2.2**	**3.8**	**4.8**
Neophytadiene	1838	2.2	1.2	3.8
Cembrene	1943		t	
Manoyloxide	1989		2.5	
13-*epi*-Manoyl oxide	1994			0.8
(*E*)-Phytol	2132		t	0.2
2-keto-manoyl oxide	2210		t	
3β-hydroxy- 13-epi- manoyl oxide	2273		0.1	
**Fatty acids and derivatives**		**24.4**	**43.3**	**10.6**
Cinnamic acid	1397			0.2
Dodecanoic acid	1566	t		
Tetradecanoic acid	1768	0.2	1.4	0.7
Pentadecanoic acid	1863		t	
Phthalic acid, diisobutyl ester	1871	0.1	1.4	4.6
(*Z,Z,Z*)-9,12-15-Octadecatrienoic acid	2099	23.5	14.7	1.7
(*Z,Z*)-9,12-Octadecadienoic acid	2122	0.6	6.6	1.4
Octadecanoic acid	2172		0.5	0.1
Eicosanoic acid	2327		2.0	0.5
Docosanoic acid	2526		2.7	
Tricosanoic acid	2628		0.4	
Tetracosanoic acid	2730		6.3	1.4
Pentacosanoic acid	2829		2.2	
Hexacosanoic acid	2934		4.3	
Heptacosanoic acid	3032		0.8	
**Phenolic compounds**		**1.4**	**1.0**	**1.2**
Carvacrol	1299	0.3	1.0	1.2
2,5-di-*tert*-butylphenol	1513	0.5		
BHT	1515	0.6		
	**R**_**t**_			
**Flavonoids**		**30.2**	**0**	**0.1**
Apigenin dimethyl ether (Genkwanin 4'-methyl ether)	43.26	5.6		
Quercetagetin 3',4',6,7-tetramethyl ether	43.36	24.6		0.1
Quercetin 3,7,3',4'-tetramethyl ether (Retusin)	47.18			
**Others**		**3.3**	**16.2**	**32.8**
Dihydroctinidiolide	19.26		0.2	0.7
Vitamin E	47.65	3.3	11.5	22.7
γ-Sitosterol	51.50			0.4
β-Amyrine	52.14		0.9	t
α-Amyrine	53.16		1.2	1.3
3β-Acetyloxyolean-12-en-28-oic acid methyl ester (Oleanolic acid methyl ester acetate)	65.18			0.5
**Total amount of compounds**		**90.1**	**92.6**	**92.2**

K_i_ : linear retention indices; R_t_: retention times; t: traces, less than 0.05%; CL: *Cistus libanotis*; CM: *Cistus monspeliensis*; CV: *Cistus villosus.*

Few reports are available in literature about the chemical constituents of *Cistus* species. The analysis of the composition of a hexane extract of leaves of *C. monspeliensis* collected in the island of Crete [7] indicated the presence of 13-*epi*-manoyl oxide, completely absent in our sample, which contains on the contrary a good quantity of manoyloxide. The available literature reports also chemical studies on the composition of extracts of *Cistus* species, obtained by different solvents. Catechin related compounds were also identified in the aqueous extracts of *Cistus monspeliensis*[8]. Some studies have reported the existence of monomeric and polymeric flavanols, gallic acid, rutin and diterpenes in several parts of *Cistus incanus*[10-12]. Previous studies have shown the presence of oligomeric proanthocyanidins in *Cistus albidus*[13]. Polyphenols in water extracts of *C. libanotis* and *C. monspeliensis* collected in Spain, have also been reported [9]. The concentration and the presence of different compounds in plants are not only species specific but they also depend on soil fertility and pH, light intensity, plant age or temperature stress [14].

The presence of flavonoids in *Cistus* has been well documented. In fact, previous reports showed the occurrence of apigenin, quercetin and kaempferol derivatives in exudates of *C. ladanifer* leaves and in soil where these plants grew [15,16], and this has been related to its allelopathic potential. In the extract of *C. libanotis* we detected as main compounds quercetin 3,7,3',4'-tetramethyl ether (retusin) (24.6%) and 5.6% of apigenin dimethyl ether (genkwanin 4'-methyl ether).

### Cytotoxic activity of the extracts

The cytotoxic activity of three *Cistus* extracts against three cancer cell lines, including murine monocyte/macrophages, J774.A1, human melanoma cells, A-375, and human breast cancer cells MCF7, was determined, through the MTT conversion assay [17]. In Table 2 we showed the IC_50_ values, that represent the concentration expressed as mg of dry extract/ml of the different hexane extracts *of C. libanotis, C. villosus* and *C. monspeliensis* that affords a 50% reduction in cell growth after 72 h incubation time. Both extracts obtained from *C. libanotis* and *C. villosus* were inactive against all tested cell lines. The 50% cytotoxic concentration (IC_50_) could not be estimated. A pronounced growth inhibition was showed by *C. monspeliensis* hexane extract against A-375 cell line, with a IC_50_ value of 82.42 ± 2.92 mg/ml at 24 h and 52.44 ± 3.69 mg/ml at 72 h. Our results indicated higher activity of *C. monspeliensis* extract if compared to 6-mercaptopurine (means IC_50_ = 142,36 mg/ml at 72h) used as reference drug (Table 2).

**Table 2:** ***In vitro *antiproliferative activity of *C. libanotis, C. monspeliensis *and *C. villosus *hexane extracts against J774.A1 macrophages, A-375 human melanoma cells and MCF-7 breast cancer cells at 72 h**

**IC**_**50**_**72 h**			
**Compound**	**J774A.1**	**MCF7**	**A375**
*Cistus libanotis*	N.D.	N.D.	N.D.
*Cistus monspeliensis*	N.D.	N.D.	52.44 ± 3.69
*Cistus villosus*	N.D.	N.D.	N.D.
6-mercaptopurine	0,003	48,23	142,36
N.D. = not detected			

IC_50_ values for different cancer cell lines are expressed in mg /mL for extracts and in μM for 6-MP, used as reference drug. The IC_50_ value is the concentration of compound that affords 50% reduction in cell growth after 3 days incubation. Values are expressed as mean ± SD, n = 3.

Natural extracts have been previously reported as a potential source of antiproliferative compounds [18-20]. In this sense, it is accepted that the chemopreventive and tumor-inhibitory effect associated to some dietary antioxidant polyphenols could be due to their capability to inhibit oxygen reactive species (ROS) or free radicals [21]. More recently, a large body of studies is evidencing the ability of these compounds to modulate uncontrolled proliferation pathways or protooncogen expression [22]. Therefore, it is certainly plausible that the antiproliferative activity against A-375 cell line of the hexane extract of *Cistus monspeliensis* compounds could be related to their radical scavenging activity too.

Phenolic compounds have been traditionally associated to biological activities such as antioxidant, antimicrobial or cytotoxic. A recent study on the anticancer activity of several tea extracts with high polyphenolic content has reported IC_50_ values within the range 0.1–0.5 mg/ml for several cancer cell lines [23].

Therefore, the concentration ranges of *C. monspeliensis* extract displaying cytotoxicity against A-375 might be significant to support further studies since hexane extract of *C. monspeliensis* is enriched in vitamin E (11.5%) which possesses well known antioxidant activity. Vitamin E acts as a peroxyl radical scavenger, preventing the propagation of free radicals in tissues, by reacting with them to form a tocopheryl radical which will then be oxidized by a hydrogen donor (such as Vitamin C) and thus return to its reduced state [24]. As it is fat soluble, it is incorporated into cell membranes and protects them from oxidative damage. The cancer preventive properties of vitamin E were firstly suspected when some studies showed that people in the Mediterranean area who consume diets enriched in vitamin E displayed a lower risk of colon cancer than people in Northern Europe and the U.S. [25,26]. More recently, the Melbourne Colorectal Cancer Study showed that dietary vitamins E and C were protective for both colon and rectal cancer, and that for both vitamins there was a dose–response effect of increasing protection [27]. Another clinical study supported a preventive effect of vitamin E in the development of prostate cancer. This study included over 29,000 elderly male smokers and showed that those taking vitamin E for six years had 32% fewer diagnoses of prostate cancer and 41% fewer prostate cancer deaths than men who did not take vitamin E [28]. More recently it has been demonstrated that Vitamin E also protects lipids and prevents the oxidation of polyunsaturated fatty acids [29].

Experimental studies also suggested detrimental effects of omega-6 polyunsaturated fatty acids (PUFA), and beneficial effects of omega-3 PUFAs on mammary carcinogenesis, possibly due to the interaction with antioxidants. Significant interactions were also found between omega-6 and long-chain omega-3 PUFAs, with breast cancer risk inversely related to long-chain omega-3 PUFAs [30]. In this light, it is interesting to note that the hexane extract of *C. monspeliensis* was represented by fatty acids (43.3%) among which the most abundant was the polyunsaturated fatty acid (*Z,Z,Z*)-9,12-15-octadecatrienoic acid (14.7%) or linolenic acid.

In this sense, *C. monspeliensis* extract could be capable to exert its antiproliferative activity by the presence of the large amounts of fatty acids and vitamin E, in the light of the available literature that reports that dietary antioxidant polyphenols are capable to inhibit reactive oxygen species or free radicals [21] and/or to modulate uncontrolled proliferation [22-24].

## Experimental

### Plant material

Leaves of *C. libanotis*, *C. monspeliensis* and *C. villosus* were collected on March 2012 from plants growing in the National Park of Boukornine (Tunisie).

### Extraction

The plant material was dried under shade and gross powdered prior to extraction. The powdered leaf (30 g) was extracted three times with 300 mL of hexane for 3 days, than the extracts were filtered through a filter paper, after that the extracts were concentrated by rotatory evaporation, and kept at 4°C until use.

### Gas chromatography

Analytical gas chromatography was carried out on a Perkin-Elmer Sigma 115 gas chromatograph fitted with a HP-5 MS capillary column (30 m × 0.25 mm i.d.; 0.25 μm film thickness). Column temperature was initially kept at 45°C for 8 min, then gradually increased to 280°C at 2.5°C min^-1^, held for 15 min and finally raised to 295°C at 10°C min^-1^. Diluted samples (1/100 v/v, in *n*-pentane) of 1 μL were injected manually at 250°C, and in the splitless mode with a 1 minute purge-off due to the small amount of oil partially utilized for biological tests. Flame ionization detection (FID) was performed at 280°C. Helium was the carrier gas (1 mL min^-1^).

### Gas chromatography - mass spectrometry

GC-MS analysis was performed on an Agilent 6850 Ser. II apparatus, fitted with a fused silica HP-1 capillary column (30 m × 0.25 mm i.d.; 0.33 μm film thickness), coupled to an Agilent Mass Selective Detector MSD 5973; ionization energy voltage 70 eV; electron multiplier voltage energy 2000 V. Mass spectra were scanned in the range 35–450 amu, scan time 5 scans/s. Gas chromatographic conditions were as reported above; transfer line temperature, 295°C.

### Identification of components

Most constituents were identified by gas chromatography by comparison of their retention indices (LRI) with either those of the literature [31,32] or with those of authentic compounds available in our laboratories. The retention indices were determined by GC-FID mode in relation to a homologous series of *n*-alkanes (C_8_-C_28_) under the same operating conditions. Further identification was made by comparison of their mass spectra on both columns with either those stored in NIST 02 and Wiley 275 libraries or with mass spectra from the literature [32,33] and our home made library. Component relative concentrations were calculated based on GC-FID peak areas without using correction factors.

### Cell lines

J774.A1 murine monocyte/macrophage, A-375 human melanoma cell line and MCF-7 human breast cancer cell line were purchased from ATCC and used to evaluate the antiproliferative activity of the hexane extracts of *Cistus* spp.. All the media and sera were purchased from Hy-Clone (Euroclone, Paignton, Devon, UK); MTT [3 (4,5-dimethylthiazol-2-yl)-2,5-phenyl-2H-tetrazolium bromide] and 6-mercaptopurine (6-MP) were from Sigma Chemicals (Milan, Italy). Cell culture was maintained at 37°C in a Hera Cell humidified CO_2_ incubator (Kandro Laboratory, Germany) with 5% CO_2_.

### MTT antiproliferative assay

Cells (J774.Al, A-375, and MCF-7 ) were harvested and suspended in complete culture media. Approximately 100 μl of the cell suspension with a concentration of 2.0×10^4^, 3.0×10^3^, 5.0×10^3^ cells, respectively were plated on 96-well microtiter plates and allowed to adhere at 37°C in 5% CO_2_ and 95% air for 24 h. Thereafter, the medium was replaced with 90 μL of fresh medium, and a 10 μL aliquot of serial dilution of each extract to test was added and the cells were incubated for tested time. Incubation was carried out for 72 h in the dark and at the end of the period, 20 μl of MTT at a concentration of 5 mg/ml was added to each well. After 3 hours of incubation, culture media in each well was aspirated and 100 μl of DMSO was added to dissolve the formazan products formed prior to recording the optical density (O.D.) at 570 nm with respect to the reference wavelength at 620 nm. In some experiments, serial dilutions of 6-MP, as reference drug, were added. The cell viability was assessed through an MTT conversion assay (Bianco *et al*., 2012). The optical density (OD) of each well was measured with a microplate spectrophotometer (Titertek Multiskan MCC/340) equipped with a 620 nm filter. The test concentration which inhibits 50% of the cell population (IC_50_) was obtained by Probit Analysis (SPSS Version 12.0.1, Chicago, IL, USA). All the experiments were carried out in triplicates and two independent experiments were performed for each test sample. The viability of each cell line in response to treatment with *Cistus* extracts was calculated as % dead cells: 100 - (OD treated/OD control) × 100.

## Conclusion

The chemical composition of the hexane extracts from the three *Cistus* species can be useful in the chemosystematics of this complex genus. The antiproliferative activity of the Cistaceae hexane extracts observed for the first time in this study against human melanoma cell line A-375 deserve further investigations in order to determine the compounds, or their combinations, which are the main responsible for antiproliferative activity and its potential mechanism.

## Findings and description of additional material

Plant material and extracts of the plants are available (MB). The GC-MS data are also available (FS).

## Competing interests

The authors declare that they have no competing interests.

## Authors’ contributions

MBJ Collected and identified the plant material, prepared the extracts and drafted the manuscript. MEK Collected and identified the plant material and drafted the manuscript. FS performed the GC-MS analysis, identified the components and drafted the manuscript. GA performed the MTT antiproliferative assay and drafted the manuscript. SM performed the MTT antiproliferative assay and drafted the manuscript. VDF identified the components and drafted the manuscript. MB prepared the extracts, identified the components and drafted the manuscript. All authors read and approved the final manuscript.
